# Induction of tumor-specific CD8^+^ cytotoxic T lymphocytes from naïve human T cells by using *Mycobacterium*-derived mycolic acid and lipoarabinomannan-stimulated dendritic cells

**DOI:** 10.1007/s00262-019-02396-8

**Published:** 2019-09-17

**Authors:** Yuji Tomita, Eri Watanabe, Masumi Shimizu, Yasuyuki Negishi, Yukihiro Kondo, Hidemi Takahashi

**Affiliations:** 1 Department of Microbiology and Immunology, Nippon Medical School, 1-1-5 Sendagi, Bunkyo-ku, Tokyo, 113-8602 Japan; 2 Department of Urology, Nippon Medical School, Tokyo, Japan

**Keywords:** CTLs, DCs, Mycolic acid, Lipoarabinomannan, BCG, *Mycobacterium tuberculosis*

## Abstract

The main effectors in tumor control are the class I MHC molecule-restricted CD8^+^ cytotoxic T lymphocytes (CTLs). Tumor-specific CTL induction can be regulated by dendritic cells (DCs) expressing both tumor-derived epitopes and co-stimulatory molecules. Immunosuppressive tolerogenic DCs, having down-regulated co-stimulatory molecules, are seen within the tumor mass and can suppress tumor-specific CTL induction. The tolerogenic DCs expressing down-regulated XCR1^+^CD141^+^ appear to be induced by tumor-derived soluble factors or dexamethasone, while the immunogenic DCs usually express XCR1^+^CD141^+^ molecules with a cross-presentation function in humans. Thus, if tolerogenic DCs can be reactivated into immunogenic DCs with sufficient co-stimulatory molecules, tumor-specific CD8^+^ CTLs can be primed and activated in vivo. In the present study, we converted human tolerogenic CD141^+^ DCs with enhanced co-stimulatory molecule expression of CD40, CD80, and CD86 through stimulation with non-toxic mycobacterial lipids such as mycolic acid (MA) and lipoarabinomannan (LAM), which synergistically enhanced both co-stimulatory molecule expression and interleukin (IL)-12 secretion by XCR1^+^CD141^+^ DCs. Moreover, MA and LAM-stimulated DCs captured tumor antigens and presented tumor epitope(s) in association with class I MHCs and sufficient upregulated co-stimulatory molecules to prime naïve CD3^+^ T cells to become CD8^+^ tumor-specific CTLs. Repeat CD141^+^ DC stimulation with MA and LAM augmented the secretion of IL-12. These findings provide us a new method for altering the tumor environment by converting tolerogenic DCs to immunogenic DCs with MA and LAM from *Mycobacterium tuberculosis*.

**Electronic supplementary material:**

The online version of this article (10.1007/s00262-019-02396-8) contains supplementary material, which is available to authorized users.

## Introduction

The most important effector cells that recognize and eliminate tumor cells are class I MHC molecule-restricted CD8^+^ cytotoxic T lymphocytes (CTLs). These CD8^+^ CTLs are mainly activated by dendritic cells (DCs) expressing both tumor-derived epitope peptide(s) presented by class I MHC molecules and by co-stimulatory molecules, such as CD40, CD80, and CD86. We have recently reported that immunosuppressive tolerogenic DCs with down-regulated co-stimulatory molecules that will inhibit tumor-specific CTL induction have been observed within tumors and seem to be induced by tumor-derived soluble factors in both mice [1] and humans [2]. However, intravenous treatment with tumor-specific MHC molecule-restricted CD8^+^ CTLs showing excellent in vitro cytotoxicity against tumors in mice has proven ineffective for tumor mass regression [3]. This may be because tolerogenic DCs inactivate the cytotoxicity of administered CTLs in vivo.

In general, peptide epitopes from externally captured antigenic proteins seem to be presented by class II MHC molecules. By contrast, internally synthesized antigenic molecules, such as viral components or tumor antigens, are presented by class I MHC molecules [4]. As shown in previous research [1, 5], it seems that most DEC-205^+^ DCs within the murine tumor mass became tolerogenic with down-regulated expression of co-stimulatory molecules despite the unique cross-presentation function of these DCs [3, 6, 7]. This function involves the CTL epitope within the externally captured antigens being presented with the class I MHC, and we have previously reported that such cross-presentation occurs after immunizing with proteins that have unique adjuvants, such as immune stimulating complexes (ISCOMs) [8] or cholera toxin [9]. Nevertheless, even if tumor-derived fragments are captured by neighboring DEC-205^+^ DCs that possess cross-presentation, and those tolerogenic DCs expressed processed tumor epitope(s) associated with class I MHCs with insufficient co-stimulatory molecules, tumor-specific CD8^+^ CTLs are unable to be primed from naïve CD3^+^ T cells in an MHC-restricted manner. However, as we have recently reported in a murine model [3], we can convert tolerogenic DEC-205^+^ DCs into immunogenic DCs in vivo using sufficient co-stimulatory molecules and sequential repetitive administration with immuno-potent lipid/glycolipids, such as α-galactosylceramide (α-GalCer), to their CD1d-molecules. Activated immunogenic DEC-205^+^ DCs within the tumor mass present tumor antigens associated with MHC-I that prime tumor-specific CD8^+^ CTLs.

Intravesical administration of live bacillus Calmette–Guerin (BCG) is considered the most potent immunotherapy against human bladder cancer (e.g., T24 bladder carcinoma) [10–12]. The proliferation of T24 cells was markedly inhibited when live BCG-infected DCs were added to the culture, while heat-killed BCG-infected DCs or uninfected DCs did not show any inhibition in vitro [13]. This may be due to the effect of cytokines, such as tumor necrosis factor (TNF)-α and interleukin (IL)-12, which are secreted from activated live BCG-infected DCs [13]. However, as shown, live BCG is toxic and more than 60% of DCs are killed when incubating for 48 h with 200 µg/mL BCG and almost 30% of DCs are eliminated even when using heat-killed BCG. Thus, we considered that replacing BCG with its non-toxic components could be useful for tumor immunotherapy. We examined the effect of two major non-toxic components of BCG, mycolic acid (MA) and lipoarabinomannan (LAM), on the activation of CD141^+^ human DCs with cross-presenting capacity [14]. MA was chosen because it can stimulate human DCs via the CD1b molecule [15] and Mincle, C-type lectin receptors [16], whereas LAM was chosen because it can activate DCs through Dectin-2, DC-SIGN, and TLR2 [17]. Using these together, we found that live toxic BCG can be substituted with non-toxic materials, such as 500 µg/mL of purified MA and 300 μg/mL LAM to activate human tolerogenic DCs within a tumor mass [1, 2].

The infectivity to human DCs is much higher in the Aoyama B isolate (the most prevalent strain of human *Mycobacterium tuberculosis* in Japan) than in BCG (an attenuated strain derived from *Mycobacterium bovis*). Given this, we examined each to compare their potency to activate CD141^+^ DCs and show that MA and LAM from the Aoyama B isolate were associated with greater potency than those from BCG. Collectively, we demonstrate how tumor-specific CD8^+^ CTLs can be induced. First, MA- and LAM-activated CD141^+^ DCs (derived from the Aoyama B isolate) are pulsed with mitomycin-C (MMC)-treated T24 tumor cells overnight. Second, they are co-cultured with unprimed naïve MHC-matched autologous T cells for an additional 14 days.

When the CD141^+^ DCs are repetitively stimulated with MA or LAM, they also secreted IL-12. However, this secretion stopped when stimulated by TLR4 through another glycolipid lipopolysaccharide (LPS). Our findings strongly suggest that we could control human tumor progression by inducing tumor-specific CD8^+^ CTLs from naïve T cells within the tumor mass through repeated administration of immuno-potent MA and LAM from *M. tuberculosis*.

## Materials and methods

### Culture medium and cell lines

The medium used for culturing immunocompetent cells, such as CTLs, was based on the RPMI 1640 complete culture medium (CCM) [18] supplemented with 10% heated FCS, 2 mM l-glutamine, 50 U/mL penicillin, 50 μg/mL streptomycin, 1 mM HEPES, 1 mM sodium pyruvate, 50 mM 2-mercaptoethanol (2-ME). Human urinary bladder carcinoma T24 cells [10] were cultured in McCoy’s 5a medium supplemented with 50 U/mL penicillin, 50 µg/mL streptomycin, and 10% FCS. Additionally, human erythromyeloblastoid leukemia K562 cells [13] were cultured in Iscove’s Modified Dulbecco’s medium supplemented with 50 U/mL penicillin, 50 mg/mL streptomycin, and 10% FCS. All items were obtained from Thermo Fisher Scientific (Waltham, MA, USA).

### Reagents

Peptidoglycans (PGN) from *Staphylococcus aureus* (Sigma-Aldrich, St. Louis, MO, USA), LPS from *Escherichia coli 026:B6* (Sigma-Aldrich), polymyxin B (Sigma-Aldrich), LAM from Aoyama B (Nakarai Tesque, Kyoto, Japan) were used for the stimulation of DCs. BCG (Tokyo 172 strain) was purchased from Japan BCG Laboratory (Tokyo, Japan). Heat-inactivated BCG was incubated for 30 min at 85 °C to kill the bacteria and other BCG samples were left at room temperature, as described below. The Aoyama B *M. tuberculosis* isolate was provided by the Research Institute of Tuberculosis/JATA, (Tokyo, Japan).

To obtain MA, both isolates were grown at 37 °C on 7H9 medium (Difco, Detroit, MI, USA) for 4 weeks and sterilized in an autoclave for 10 min at 121 °C; the sterilized bacterial cells were collected by centrifugation. To extract lipids, cells were sonicated and extracted with chloroform/methanol (3:1 and 2:1, v/v). MA were liberated by alkali hydrolysis from the chloroform/methanol residues [19]. After methylation with benzene/methanol/H_2_SO_4_ (10:20:1, v/v) at 70 °C for 3 h, each subclass of α-, methoxy-, and keto-mycolic acid methyl esters was separated by thin-layer chromatography of silica gel (Merck Millipore, Burlington, MA, USA), developed with the solvent system benzene (Kanto Chemical, Tokyo, Japan).

### Cells

Peripheral blood mononuclear cells (PBMCs) were freshly isolated from the peripheral blood of healthy volunteers using Ficoll-Hypaque (Amersham-Pharmacia Biotech, Uppsala, Sweden). CD3^+^ T cells were separated by magnetic depletion using a negative isolation kit (BioLegend, San Diego, CA, USA) and CD14^+^ monocytes were separated by magnetic depletion using a monocyte isolation kit (STEMCELL, Vancouver, BC, Canada), each according to the manufacturer’s instructions. To obtain monocyte-derived DCs (MDDCs), 5 × 10^5^ CD14^+^ cells were cultured in 24-well plates for 6 days in 1 mL of CCM supplemented with 100 ng/mL GM-CSF (PeproTech, Rocky Hill, NJ, USA) and 10 ng/mL IL-4 (PeproTech). To assess DC stimulation, 1 × 10^5^ DCs were incubated in 200 µL CCM in 48-well plates for 2 h with live BCG, heat-inactivated BCG, MA, LAM, or LPS. After washing in buffer, cells were incubated for 48 h at 37 °C before cells and their supernatants were collected. The cell populations, surface marker expressions, and IL-12p40 concentrations were analyzed by flow cytometry or enzyme-linked immunosorbent assay (ELISA). To obtain T24 cell-induced tolerogenic DCs, 1 × 10^5^ T24 cells in trans-well were co-cultured with 5 × 10^5^ CD14^+^ cells in a 24-well plate for 6 days. To obtain tolerogenic DCs induced by dexamethasone (DEX) (Sigma-Aldrich), 5 × 10^5^ MDDCs were plated in a 24-well plate in the presence of 1 mL CCM and 1 µg/mL DEX for 24 h [20].

### Antibodies and flow cytometric analysis

The following antibodies were purchased from BioLegend: CD11c-PE/Dazzle 594 (N418), CD40-PE/Cy7 (5C3), CD141-BV421(M80), and XCR1-PE (S15046E), TLR2-PE (TL2.1). In addition, the following were purchased from BD Biosciences (San Diego, CA): CD1a-PE (HI149), CD1b-FITC (M-T101), CD80-BV605 (L307.4), CD83-BUV737 (HB15e), and CD86-BV421 (2331). For dead cell discrimination, cells were treated with a Zombie Aqua Fixable Viability Kit (BioLegend). Nonspecific binding was blocked using 10 µg human immunoglobulin polyglobin (Nippon Red Cross, Tokyo, Japan). Cells were stained with the relevant antibodies at 4 °C for 30 min in FACS buffer (phosphate-buffered saline (PBS) with 2% FCS and 10 mM sodium azide), washed twice, and resuspended in a FACS buffer. Labeled cells were then analyzed with an LSR Fortessa X-20 (BD Biosciences), using FlowJo Software (BD Biosciences). Supplementary Fig. a shows the Gating strategy for obtaining cells.

### Blocking of MDDC function by various antibodies

To block MDDC function [21], we incubated MDDCs with anti-TLR2 (TL2.1) (BioLegend), anti-TLR4 (HTA125) (BioLegend), anti-Mincle (1H2) (MBL, Nagoya, Japan), anti-DC-SIGN (AZND1) (BECMAN COULTER, Brea, CA, USA), or anti-Dectin-2 (Q7-4B5) (Invitrogen, San Diego, CA, USA) for 30 min at 37 °C. After washing in buffer, MDDCs were stimulated by PGN, MA, and LAM, as described above, and we measured CD86 expression by flow cytometry.

### Protein staining by sodium dodecyl sulfate–polyacrylamide gel electrophoresis

We mixed 10 µg MA and LAM with SDS, and 1 µg PGN and LPS with SDS and dithiothreitol. The samples were denatured at 70 °C for 10 min before being loaded onto a 12% SDS-PAGE gel (ATTO, Tokyo, Japan). After electrophoresis, protein bands were visualized by staining with a silver stain kit (GE Healthcare, Uppsala, Sweden).

### Short interfering RNA transfection

Short interfering RNA (siRNA) targeting human CD1b (ON-TARGET plus siRNA, identifier: J-01499-05, 06, 07 and 08) and control siRNA (identifier: D-001810-10-20 and D-001810-02-05) were obtained from Dharmacon (Horizon Discovery, Cambridge, UK). Briefly, MDDCs were pulsed with siRNAs (final 500 mM) at 250 V and 950 µF using a Gene Pulser II apparatus (Bio-Rad Laboratories, Hercules, CA, USA).

### Quantification of mRNA by real-time PCR

We determined mRNA levels by quantitative real-time PCR, using a commercial kit (TaqMan Gene Expression Master Mix: Thermo Fisher Scientific). Briefly, 5 μL of Master Mix, 0.5 μL TaqMan Assay (Thermo Fisher Scientific), and 2.5 μL nuclease-free water were mixed and 2 μL of cDNA (corresponding to 5 ng of the RNA template) was added to the reaction mixture. The reaction was executed by PIKOREAL96 (Thermo Fisher Scientific). The TaqMan Assay IDs were Hs009575378_m1 (CD1b) and Hs02786624_gl (GAPDH). The relative expression was calculated by the equation 2^(−Δ cycle threshold [ΔCt] × 1000)^. The ΔCt value was calculated by subtracting the Ct value (target gene − internal control gene), where GAPDH was the internal control.

### Measurement of cytokine production by ELISA

IL-10 and IL-12p40 production in the cultured supernatant were measured with ELISA kits (BioLegend).

### DC uptake, processing, and presentation of tumor antigens derived from MMC-treated T24 cells

We treated 1 × 10^7^ T24 bladder tumor cells (HLA-A1^+^ and HLA-A3^+^) [10] with MMC (Kyowa Hakko-Kirin, Tokyo, Japan) at 37 °C for 1 h, washed twice with culture medium, and further incubated at 37 °C overnight in culture medium. MMC-treated T24 cells were stained with an Allophycocyanin Annexin V apoptosis detection kit containing 7-Amino-Actinomycin D (BioLegend), and their apoptotic state was analyzed by flow cytometry. The T24 tumor antigen on MMC-treated T24 cells was then labeled with 1 µM PKH67 (Sigma-Aldrich) and 1 × 10^5^ of the labeled T24 cells were co-cultured with an equal number of commercially obtained HLA-matched DCs (HLA-A1^+^ and HLA-A3^+^) at 37 °C overnight. To see whether DCs can present captured tumor antigens expressing PKH67, co-cultured cells were further stained with CD1a and CD141, before being analyzed by flow cytometry.

### Priming and induction of tumor-specific CTLs

In the next step, we assessed the priming and induction of tumor-specific CTLs from naïve HLA-matched CD3^+^ T cells by MA- and LAM-activated HLA-matched DCs expressing T24 tumor antigens. To do so, 5 × 10^5^ HLA-matched DCs (HLA-A1^+^ and HLA-A3^+^) were incubated in 1 mL of CCM in 24-well plates for 24 h with 500 µg/mL MA and/or 300 µg/mL LAM, before being co-cultured with 1 × 10^5^ MMC-treated T24 cells at 37 °C overnight. The MA and/or LAM-activated HLA-matched DCs were irradiated with 30 Gy. Next, 1 × 10^6^ HLA-matched CD3^+^ T cells (HLA-A1^+^ and HLA-A3^+^) were added to the culture containing 2 × 10^5^ MA- and/or LAM-activated HLA-matched DCs expressing T24 tumor antigen, and these were further incubated for 2 weeks in 1 mL of CCM supplemented with 20 U/mL IL-2 (Shionogi, Osaka, Japan). Two weeks later, 2 × 10^5^ MA and/or LAM-activated HLA-matched DCs expressing T24 tumor antigen were further cultured for an additional 2 weeks in 1 mL of CCM to obtain effector T cells. In some experiments, the effector T cells were treated with 10 µg/mL anti-HLA-ABC (BioLegend), and in others, CD8^+^ T cells were depleted by using an anti-CD8^+^ T cells selection kit (BioLegend) and their cytotoxicities were examined.

### Chromium-51 (^51^Cr)-release assay

The cytotoxicity of effector T cells was measured by a standard 4-h ^51^Cr-release assay that used T24 cells, K562 cells, or tumor antigen-pulsed DCs as targets. Effector cells were incubated with ^51^Cr-labeled target cells (5 × 10^3^) for 4 h at 37 °C in 200 µL of RPMI 1640 medium containing 10% FCS in round-bottomed 96-well cell culture plates. After incubation, the plates were centrifuged for 10 min at 330×*g*, and 100 µL of cell-free supernatant was collected to measure radioactivity with a Wallac Wizard 3 (1480 Automatic Gamma Counter; Perkin Elmer, Waltham, MA, USA). Maximum release was determined from the supernatant of cells that had been lysed by the addition of 5% Triton X-100, and spontaneous release was determined from target cells incubated without added effector cells. The percent specific lysis was calculated as follows: 100 × (experimental release − spontaneous release)/(maximum release − spontaneous release).

### MLR assays

CD4^+^ T cells were selected from PBMCs by the CD4^+^ T cell isolation kit (BioLegend). We co-cultured 2 × 10^4^ CD4^+^ T cells with 4 × 10^2^ irradiated allogenic antigen-presenting cells (APCs) (either untreated, in T24 co-culture, or with DEX) in 96 well plates for 6 days. Cell proliferation was assessed by Cell Counting Kit-8 (Dojindo, Tokyo, Japan).

### IL-12p40 production by MDDCs after sequential PGN, LPS, MA, and LAM stimulation

In brief, 5 × 10^5^ DCs in 1 mL of CCM in 24-well plates were stimulated by PGN (10 µg/mL), LPS (100 ng/mL), MA (500 µg/mL), or LAM (300 µg/mL) for 2 h. After washing in buffer, MDDCs were incubated for 48 h (first stimulation) and then stimulated again by ligands for 2 h before being incubated again for 48 h (second stimulation). We repeated this stimulation of MDDCs by ligands three times. After each incubation, the IL-12p40 concentration in the supernatant was determined by ELISA. The experimental setup is shown in Fig. 6h.

### Statistical analyses

All statistical analyses were performed using Prism (GraphPad Software, SanDiego, CA, USA). For comparisons between more than two groups, we used one-way analysis of variance (ANOVA) followed by Bonferroni post-tests. For comparisons between more than two groups, when there were two independent variables (Fig. 6i), we used two-way ANOVA followed by Bonferroni post-tests. Results are presented as mean ± standard deviation (SD). A *p* value < 0.05 was taken to indicate a statistically significant difference.

## Results

### Effect of BCG-treatment on MDDCs

The main targets for *M. tuberculosis* (e.g., BCG) are DCs, among which CD141^+^ cells are known to retain cross-presenting epitopes within captured antigenic molecules via class I MHC. This also applies to co-stimulatory molecules, such as CD40, CD80, and CD86, which will assist to prime and activate CD8^+^ epitope-specific CTLs. Activated CTLs will then attack cells expressing the epitope(s) presented with the class I MHC molecules.

**Fig. 1:**
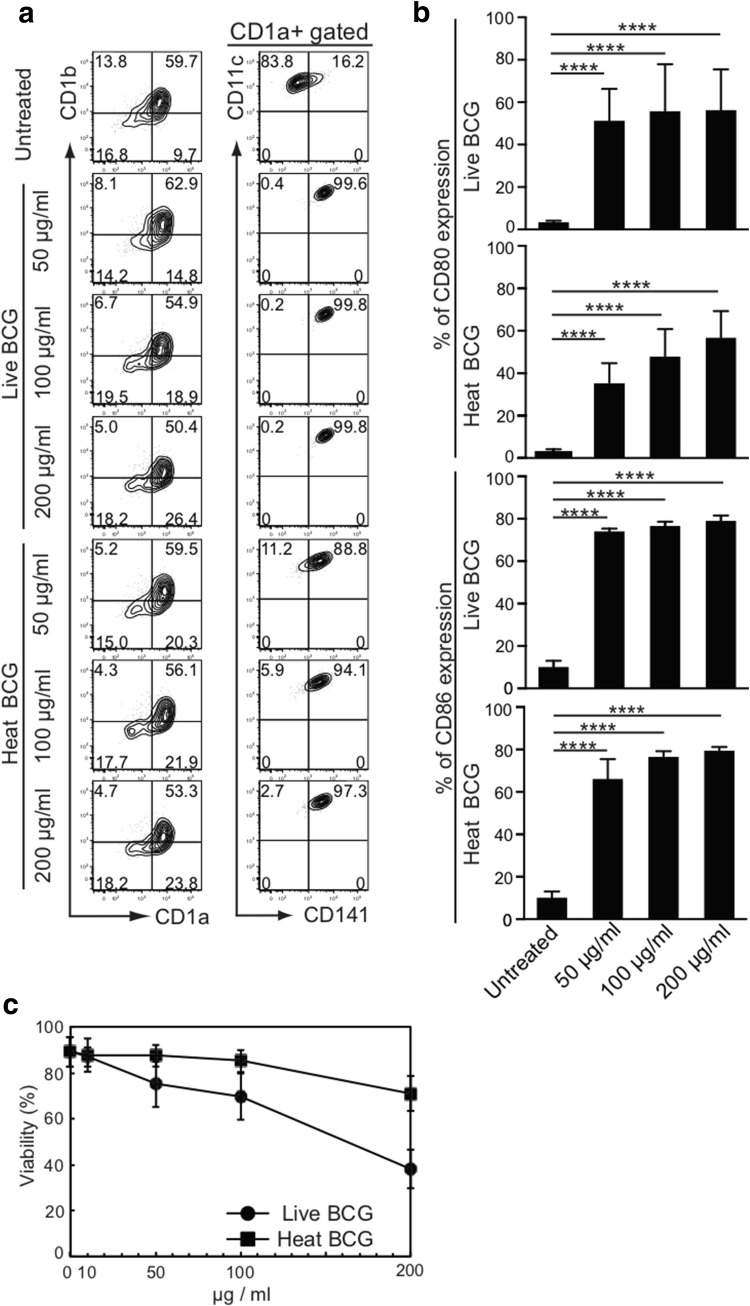
Effect of BCG-treatment on MDDCs. **a** Representative FACS plots of CD1a, CD1b, CD141, and CD11c expression on viable cells stimulated with live and heat-inactivated BCG. **b** Alteration of CD80 and CD86 expressions on MDDCs. Data are shown as the mean ± SD of *n* = 6 samples per measurement. ****p < 0.0001; one-way ANOVA followed by Bonferroni post-tests. **c** Viability of MDDCs co-cultured with live or heat-inactivated BCG

To examine whether BCG could selectively activate CD141^+^ DCs, we incubated MDDCs in CCM [18] that contained various doses of either live or heat-inactivated BCG for 2 h. These were then washed extensively to remove free BCG and further incubated for an additional 2 days in BCG-free CCM and CD141^+^ expression was observed. The percentage of CD141^+^CD11c^+^ DCs was significantly increased among the DCs treated with both live and heat-inactivated BCG, although the activated percentage was slightly higher among the group treated with live BCG (right lane, Fig. 1a). However, the percentage of CD1a^+^CD1b^+^ DCs markedly declined when more than 200 µg/mL of live BCG was used for the treatment (left lane, Fig. 1a). We thought that the decline was caused by the toxicity of live BCG. Among CD141^+^CD11c^+^ DCs, CD80 and CD86 expression were both increased dose dependently (Fig. 1b). Thus, we compared the viability of DCs after treatment with various doses of live and heat-inactivated BCG. As expected, the number of viable DCs declined sharply as the amount of BCG increased (Fig. 1c). Indeed, more than 60% of DCs were dead after incubation with 200 µg/mL of live BCG for 2 d (Fig. 1c). Additionally, it should be noted that near 30% of DCs were killed after the same incubation period with 200 µg/mL of heat-inactivated BCG (Fig. 1c).

Not only live BCG but also heat-inactivated BCG appeared to be toxic to DCs that are critical for cancer immunotherapy. Moreover, although intravesical BCG therapy has been widely considered the most successful immunotherapy against human bladder carcinoma [22], various adverse events exist, such as tuberculous nephritis, severe cystitis, and gross hematuria, caused by the BCG subcomponent [23–25]. These adverse effects seem to be associated with the cellular damage initiated by live and heat-inactivated BCG. Thus, whole BCG, even attenuated, could be toxic and should be avoided in such treatment.

### Possible PGN and LPS contamination within purified MA and LAM

**Fig. 2:**
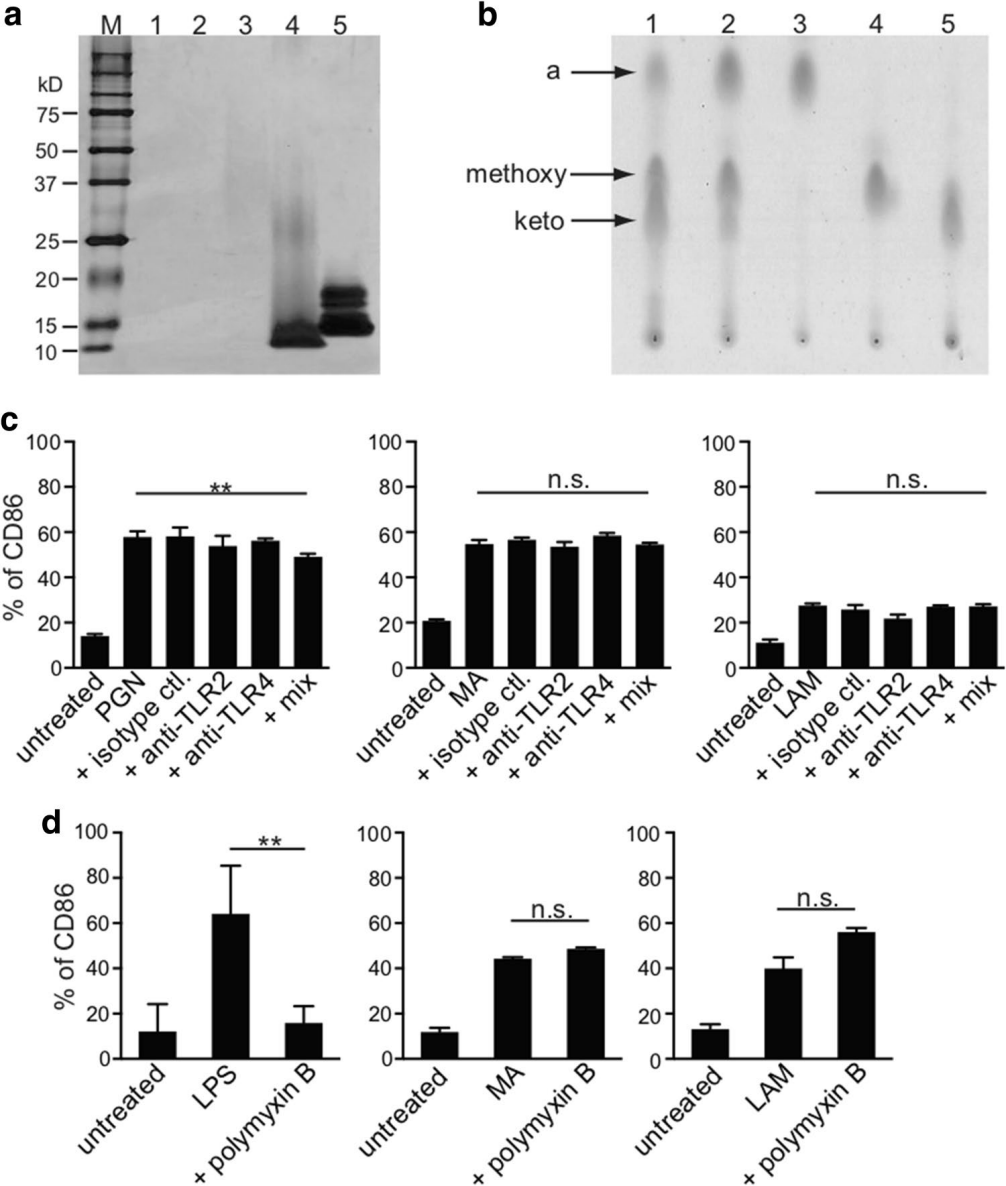
Confirmation of MA and LAM purity. **a** Protein detection by silver stain SDS-PAGE gel. Lane 1: 10 µg MA from BCG isolate, Lane 2: 10 µg MA from Aoyama B isolate, Lane 3: 10 µg LAM from Aoyama B isolate, Lane 4: 1 µg PGN from *Staphylococcus aureus*, Lane 5: 1 µg LPS from *Escherichia coli*. Numbers shown in the left-axis indicate molecular weights (kD). **b** Thin-layer chromatography of purified MA. Lane 1: MA from BCG, Lane 2: MA from Aoyama B isolate, Lane 3: α-MA from Aoyama B isolate, Lane 4: methoxy-MA from Aoyama B isolate, Lane 5: keto-MA from Aoyama B isolate. All samples were 50 µg. **c** CD86 expression on MDDCs stimulated by 10 µg/mL PGN (left), 200 µg/mL MA from Aoyama B isolate (middle), 200 µg/mL LAM (right) treated with anti-TLR2/4 antibodies. Data are shown as the mean ± SD of *n *= 4 samples per measurement. **d** CD86 expression on MDDCs stimulated by 100 ng/mL LPS (left), 200 µg/mL MA from Aoyama B isolate (middle), 200 µg/mL LAM (right) with or without 15 µg/mL polymyxin B. Data are shown as the mean ± SD of *n *= 4 samples per measurement. **p < 0.01, *n.s*. not significant; one-way ANOVA followed by Bonferroni post-tests

We then examined the purity of non-toxic BCG components, such as MA and LAM. First, we investigated the possibility of protein contamination within purified MA and LAM samples, using silver staining SDS-PAGE gel, as shown in Supplementary Fig. b. In comparison with the positive controls indicated in lane 4 (PGN from *Staphylococcus aureus*) and lane 5 (LPS from *Escherichia coli*), we detected no protein contamination in the purified MA from the BCG or Aoyama B isolate; however, we detected a subtle amount of protein within the purified LAM from the Aoyama B isolate (lane 3) (Fig. 2a). We also detected no lipid contamination in the purified MA when using thin-layer chromatography. MA from BCG (lane 1), MA from Aoyama B (lane 2), α-MA from Aoyama B (lane 3), methoxy-MA from Aoyama B (lane 4), and keto-MA from Aoyama B (lane 5) are shown, and more α-MA and methoxy-MA than keto-MA was detected in the Aoyama B isolate, while less α-MA was observed in BCG (Fig. 2b). Although enhanced CD86 expression was seen within the activated MDDCs when stimulated with either 10 µg/mL PGN (left), 200 µg/mL MA from Aoyama B (middle), or 200 µg/mL LAM (right), enhancement was not suppressed by anti-TLR2/4 antibody treatment, indicating that PGN was not contaminated in the purified MA and LAM (Fig. 2c). In addition, although enhanced CD86 expression on MDDCs stimulated with LPS was totally abrogated by polymyxin B (left panel of Fig. 2d), CD86 enhancement induced with both MA and LAM was not inhibited (middle and right panels of Fig. 2d), suggesting that purified MA and LAM were not contaminated with LPS. Therefore, we can disregard the potential for contamination with PGN and LPS within the purified MA or LAM samples.

### Effect on MDDC activation of purified MA and LAM from *M. tuberculosis*

**Fig. 3:**
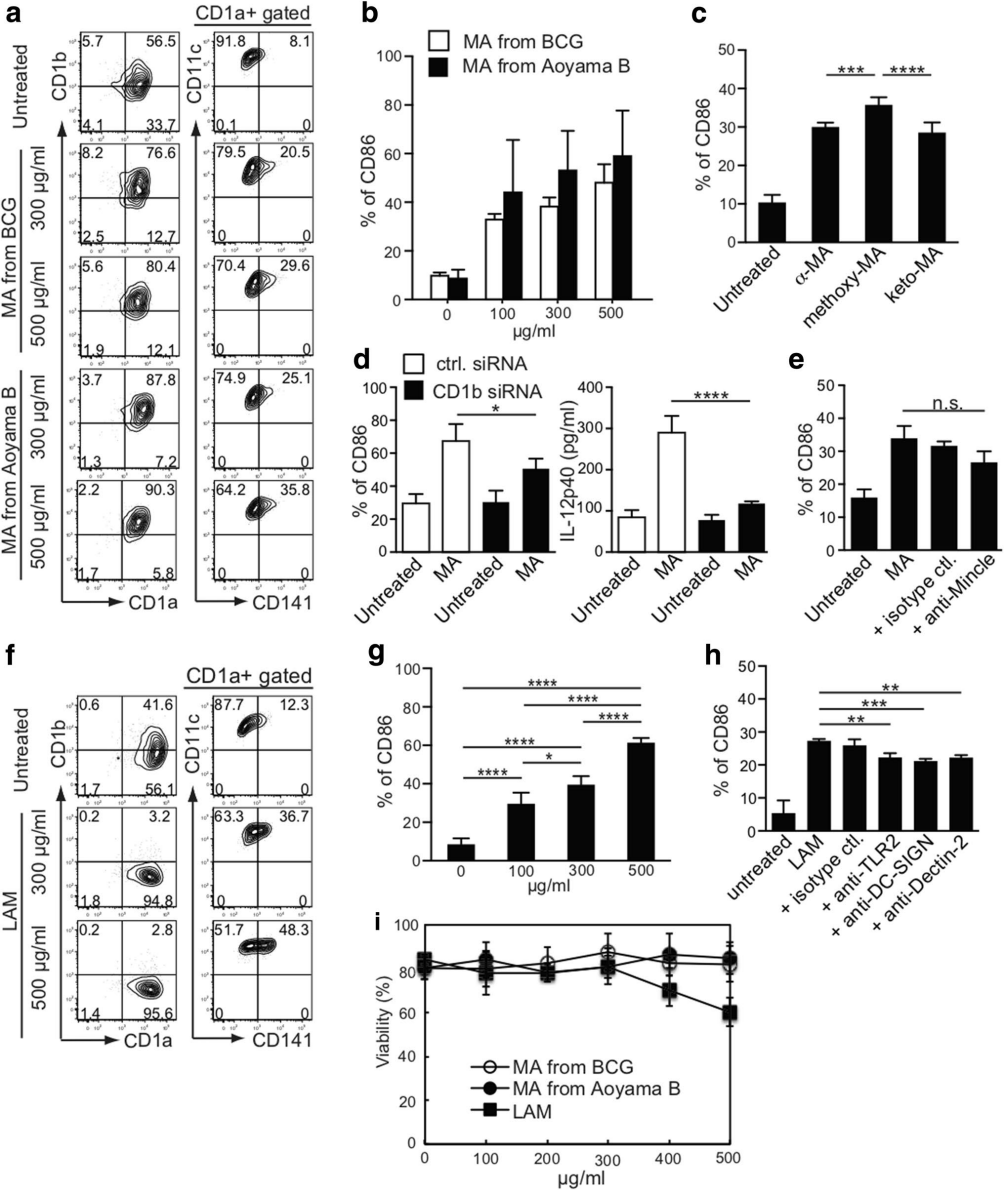
Effect of purified non-toxic MA and LAM treatment of MDDCs on the expression of co-stimulatory molecules. **a** Representative FACS plots of CD1a, CD1b, CD141, and CD11c expression on viable cells stimulated with MA from the BCG and Aoyama B isolate. **b** CD86 expression on MDDCs stimulated with MA from BCG and Aoyama B isolate. Data are shown as the mean ± SD of *n* = 4 samples per measurement. **c** CD86 expression on MDDCs stimulated with 500 µg/mL of α-MA, methoxy-MA, and keto-MA among Aoyama B isolate which showed stronger stimulatory potency than BCG. Data are shown as the mean ± SD of *n* = 6 samples per measurement. **d** CD86 expression and IL-12p40 production on CD1b-siRNA-MDDCs stimulated by 200 µg/mL MA from Aoyama B isolate. Data are shown as the mean ± SD of *n *= 4 samples per measurement. **e** CD86 expression on MDDCs stimulated by 200 µg/mL MA from Aoyama B isolate treated with anti-Mincle antibody. Data are shown as the mean ± SD of *n *= 3 samples per measurement. **f** Representative FACS plots of CD1a, CD1b, CD141, and CD11c expression on viable cells stimulated with LAM. **g** CD86 expression on MDDCs stimulated with LAM. Data are shown as the mean ± SD of *n* = 4 samples per measurement. **h** CD86 expression on MDDCs stimulated by 200 µg/mL LAM treated with anti-TLR2, anti-DC-SIGN, or anti-Dectin-2 antibodies. **i** Viability of MDDCs stimulated with MA from BCG, MA from Aoyama B isolate, and LAM. *p < 0.05, **p < 0.01, ***p < 0.001, ****p < 0.0001; one-way ANOVA followed by Bonferroni post-tests

As reported, we detected more α-MA and methoxy-MA than keto-MA in the Aoyama B isolate, but less α-MA in BCG (Fig. 2b). Moreover, CD141^+^ expression was more remarkably enhanced when DCs were treated with MA purified from the Aoyama B isolate than with MA from BCG, and this occurred in a dose-dependent manner (Fig. 3a). MA from Aoyama B, rather than from BCG, enhanced CD86 expression on MDDCs (Fig. 3b). Furthermore, methoxy-MA from the Aoyama B isolate most effectively enhanced CD86 expression on MDDCs (Fig. 3c). Based on recent findings [26], we examined CD86 expression and IL-12p40 production in MDDCs with down-modulated CD1b by using CD1b-siRNA transfected-MDDCs and confirmed the downmodulation (Fig. 3d; Supplementary Fig. c). This clearly indicated that we cannot apply the mouse model when using MA because mice do not express CD1b. The anti-Mincle antibody also inhibited CD86 expression on MDDCs stimulated by MA from the Aoyama B isolate (Fig. 3e). When the DCs were treated with LAM (500 or 300 μg/mL), CD141^+^ and CD86 expressions were both enhanced (Fig. 3f, g). As expected, the CD86 expression induced by LAM was reduced by treatment with either anti-TLR2, anti-DC-SIGN, or anti-Dectin-2 antibody (Fig. 3h). It should be noted that a purified LAM dose exceeding 400 µg/mL was toxic to DCs (Fig. 3i). Thus, 500 µg/mL MA from the Aoyama B isolate and 300 µg/mL of LAM was non-toxic and effective for activating DCs.

### Effect of purified MA and LAM on the expression of co-stimulatory molecules and on IL-12-production

**Fig. 4:**
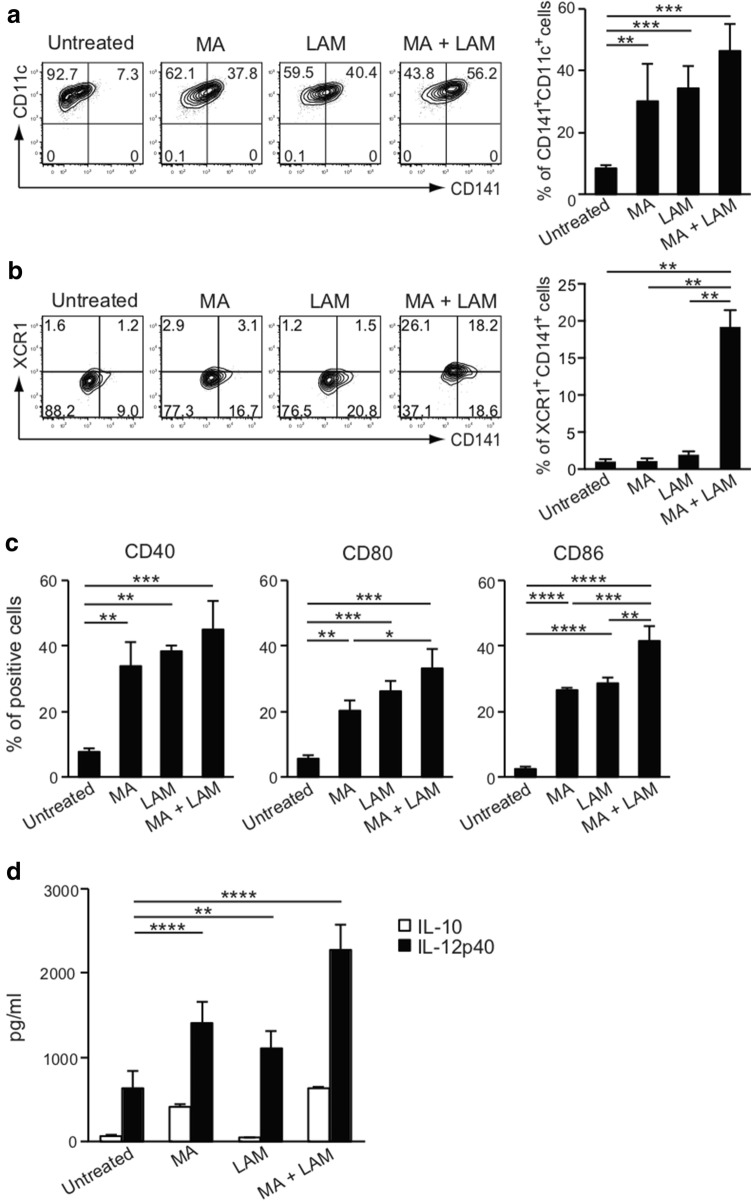
Effect of MA and LAM treatment on MDDCs. **a** Percentage of CD141^+^ CD11c^+^ cells among CD1a^+^ viable cells stimulated with MA and/or LAM from Aoyama B isolates was measured by flow cytometry. Data are shown as the mean ± SD of *n* = 3 samples per measurement. **b** The percentage of XCR1^+^CD141^+^ cells stimulated with MA and/or LAM from Aoyama B isolates was measured. Data are shown as the mean ± SD of *n* = 4 samples per measurement. **c** Expression of co-stimulatory molecules on MDDCs stimulated with MA and/or LAM from Aoyama B isolates. Data are shown as the mean ± SD of *n* = 3 samples per measurement. **d** IL-10 and IL-12p40 production of MDDCs stimulated with MA and/or LAM from Aoyama B isolate in supernatant. Data are shown as the mean ± SD of *n* = 4 samples per measurement. *p < 0.05, **p < 0.01, ***p < 0.001, ****p < 0.0001; one-way ANOVA followed by Bonferroni post-tests

*Mycobacterium*-derived purified MA and LAM subcomponents at appropriate dosages are both non-toxic for DCs and have a potent ability to enhance the CD141^+^ expression. Both MA and LAM therefore markedly augmented the cross-presenting ability of the captured proteins through class I MHC molecules. The activated CD141^+^ DCs then induce epitope-specific CD8^+^ CTLs in tandem with co-stimulatory molecules, such as CD40, CD80, or CD86. Thus, we have demonstrated the effect of MA and/or LAM on co-stimulatory molecules. Both MA- and LAM-stimulation enhanced CD141^+^CD11c^+^ expression (Fig. 4a) and XCR1^+^CD141^+^ expression (Fig. 4b) on MDDCs. Moreover, MA and LAM synergistically enhanced co-stimulatory molecules expressed on CD141^+^ DCs (Fig. 4c) and also enhanced IL-12 secretion, which is a critical cytokine for inducing CTL (Fig. 4d). Therefore, MA- and LAM-treated DCs may gain the ability to induce antigen-specific CTLs.

### MA and/or LAM from the DCs activated by the Aoyama B isolate can prime tumor-specific class I MHC-restricted human CD8^+^ CTLs

**Fig. 5:**
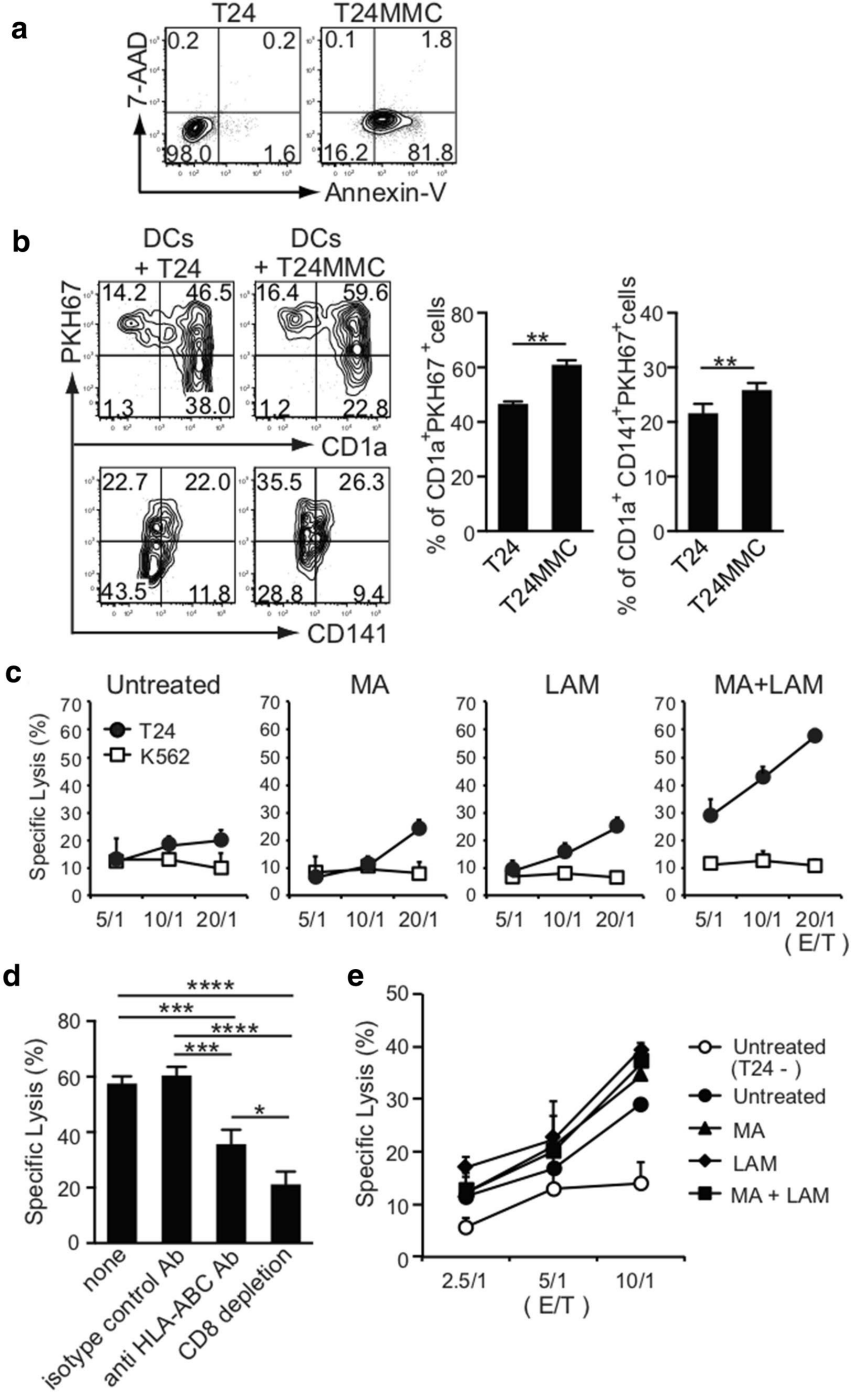
Priming of tumor-specific class I MHC-restricted human CD8^+^ CTLs by MA and/or LAM from the Aoyama B isolate-activated MDDCs. **a** Apoptosis of T24 cells treated with MMC was evaluated by flow cytometry using Annexin V and 7-Amino-Actinomycin D. **b** Percentage of CD1a^+^ PKH67^+^ cells and CD1a^+^ CD141^+^ PKH67^+^ cells in MDDCs co-cultured with T24 cells with/without MMC. Data are shown as the mean ± SD of *n* = 5 samples per measurement. **c** Specific lysis of T24 cells and K562 cells by CTLs stimulated by MHC-matched MDDCs co-cultured with MA and/or LAM. **d** Specific lysis of T24 cells by effector T cells treated with anti-HLA-ABC antibody or depleted of T24-specific CD8^+^ T cells. Graph shows the percent of specific lysis at effector-to-target cell ratio (20/1). Data are shown as the mean ± SD of *n* = 3 samples per measurement. **e** Specific lysis of tumor antigen-presenting DCs co-cultured with MA and/or LAM. *p < 0.05, **p < 0.01, ***p < 0.001, ****p < 0.0001; one-way ANOVA followed by Bonferroni post-tests

We examined whether MA- or LAM-treated CD141^+^ DCs loaded with tumor antigen can gain the ability to prime naïve autologous CD8^+^ T cells into tumor-specific CTLs in a class I MHC-restricted manner by cross-reactivity. Human T24 bladder tumor cells expressing HLA-A1 and -A3 were first incubated with MMC at 37 °C overnight to confirm that they became apoptotic, using a flow cytometric analysis and Annexin V staining (Fig. 5a). The apoptotic T24 cells stained in PKH67 green were cultured with DCs expressing HLA-A1 and -A3 purchased commercially from a donor to confirm whether DCs captured apoptotic T24 fragments (Fig. 5b). Then, MHC-matched DCs were incubated with 500 µg/mL MA and/or 300 µg/mL LAM and were co-cultured with MMC-treated T24 cells overnight at 37 °C before being further co-cultured with MHC-matched naïve CD3^+^ T cells MA for an additional 2 weeks in the presence of IL-2 and MMC-treated T24 tumor cells. Strikingly, we found that T24 tumor cells were specifically and strongly killed by the CD8^+^ T cells induced by MHC-matched DCs pre-treated with both MA and LAM, even though these DCs showed some cytotoxicity (Fig. 5c). It should also be noted that the T24-specific CTL cytotoxicity was markedly suppressed by treatment with either HLA-ABC-specific antibody or CD8 T^+^ cell depletion (Fig. 5d).

Tumor-specific, class I MHC-restricted CD8^+^ CTLs also eliminated ^51^Cr-labeled MHC-matched DCs almost equally (Fig. 5e). This indicated that DCs preincubated overnight with apoptotic T24 cells expressed the T24 tumor antigen through their class I MHC molecules (e.g., HLA-A1 and -A3). These findings indicate that MA- and LAM-treated DCs can gain the ability to cross-present captured tumor antigens via class I MHCs and can prime and activate neighboring HLA-matched naïve CD8^+^ T cells into tumor-specific CD8^+^ CTLs.

### Establishment of tolerogenic human CD141^+^ DCs expressing down-modulated co-stimulatory molecules

**Fig. 6:**
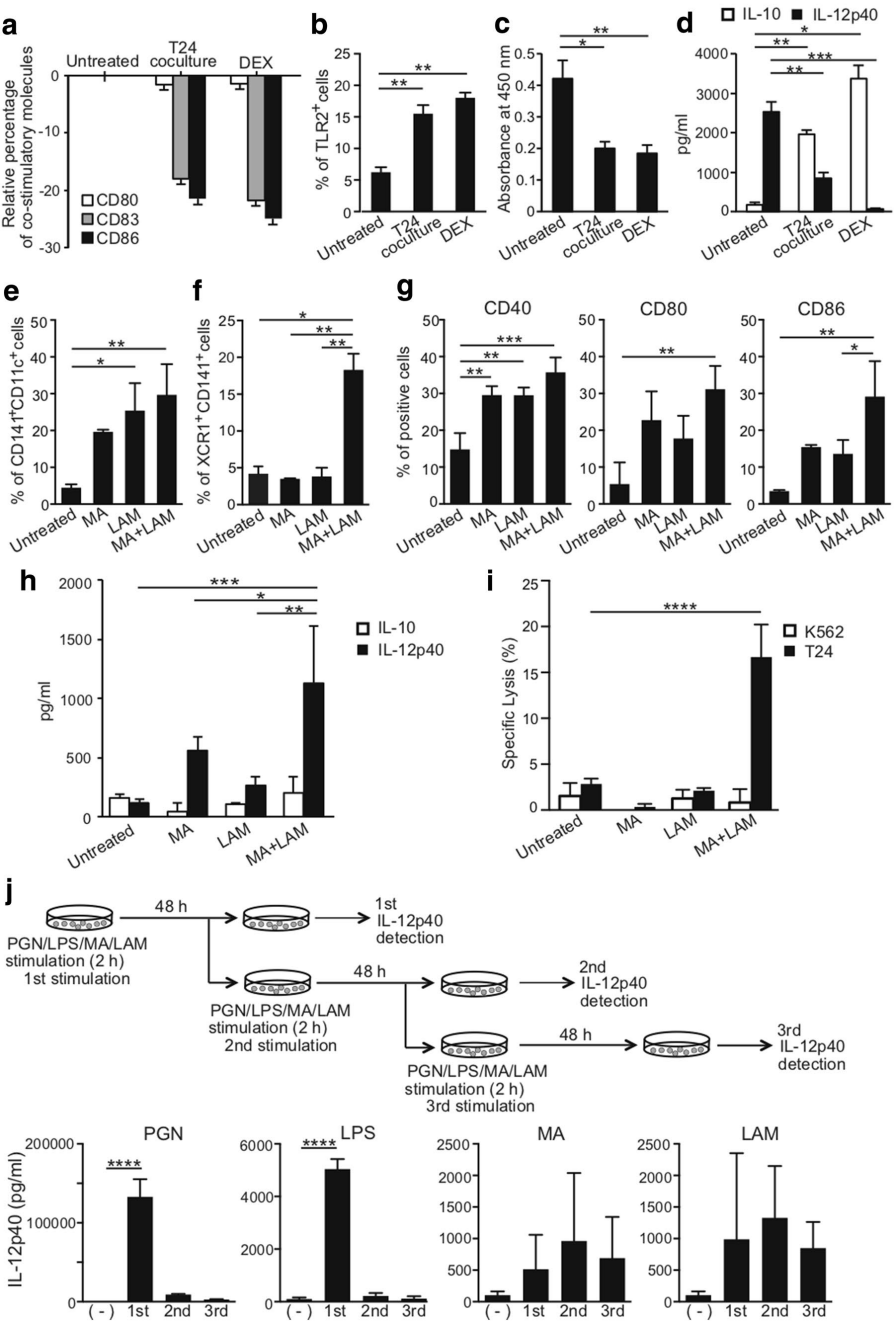
Conversion of tolerogenic DCs to immunogenic DCs by MA and LAM. **a** Relative percentage of co-stimulatory molecules, CD80, CD83, and CD86 on MDDCS treated by T24 co-culture and DEX to untreated cells. Data are shown as the mean ± SD of *n* = 4 samples per measurement. Data **b** percentage of TLR2 expression on tolerogenic DCs. Data are shown as the mean ± SD of *n* = 4 samples per measurement. **c** Absorbance of proliferated CD4 + T cells by mixed lymphocyte reaction. Tolerogenic DCs were cultured with allogeneic CD4 + T cells for 6 days. Data are shown as the mean ± SD of *n* = 4 samples per measurement. APC-to-T cell ratio (50/1). **d** IL-10 and IL-12p40 production was measured by ELISA in supernatants harvested after 10 ng/mL LPS stimulation. Data are shown as the mean ± SD of *n* = 4 samples per measurement. **e** Percentages of CD141 and CD11c expression on immunogenic DCs. Tolerogenic DCs induced by T24 cells were converted to immunogenic DCs with MA and/or LAM. Data are shown as the mean ± SD of *n* = 3 samples per measurement. **f** Percentages of XCR1 and CD141 expression on immunogenic DCs. Data are shown as the mean ± SD of *n* = 3 samples per measurement. **g** Up-regulation of CD40, CD80, and CD86 on immunogenic DCs. Data are shown as the mean ± SD of *n* = 3 samples per measurement. **h** IL-10 and IL-12p40 production by immunogenic DCs in the supernatant. Data are shown as the mean ± SD of *n* = 4 samples per measurement. **i** Specific lysis of T24 cells and K562 cells by CTLs activated by MHC-matched immunogenic DCs. Graph shows the percent of specific lysis at effector-to-target cell ratio (20/1). Data are shown as the mean ± SD of *n* = 3–4 samples per measurement. **j** IL-12p40 production by MDDCs with sequential stimulation by 10 µg/mL PGN, 100 ng/mL LPS, and 500 µg/mL MA from the Aoyama B isolate, and 300 µg/mL LAM every 48 h. *p < 0.05, **p < 0.01, ***p < 0.001, ****p < 0.0001; one-way ANOVA or two-way ANOVA followed by Bonferroni post-tests

We have established human tolerogenic DCs by co-culturing MDDCs with T24 tumor-derived factors or DEX [20, 27]. Indeed, we obtained tolerogenic DCs with down-regulated co-stimulatory molecules, such as CD80, CD83 and CD86, in this research (Fig. 6a). In addition, TLR2 expression was augmented (Fig. 6b) while the allogeneic T cell response was suppressed (Fig. 6c) and IL-10 secretion was increased, despite a reduction of IL-12 secretion in tolerogenic DCs (Fig. 6d). By contrast, incubation of tolerogenic DCs with MA and LAM converts them to immunogenic DCs, with greater numbers of CD141^+^CD11c^+^ DCs (Fig. 6e), XCR1^+^CD141^+^ DCs (Fig. 6f), and co-stimulatory molecules (Fig. 6g). Moreover, MA- and LAM-stimulated DCs secreted more IL-12 despite not secreting IL-10 (Fig. 6h), and MA and LAM stimulated the CD141^+^CD11c^+^ induced T24-specific CTLs (Fig. 6i).

### Induction of CD141^+^ immunogenic DCs by MA and LAM

In previous research [3], we demonstrated that sequential and repetitive inoculation with intraperitoneal α-GalCer every 48 h appeared to convert tolerogenic DEC-205^+^ DCs into immunogenic DCs with a higher expression of co-stimulatory molecules and stronger cross-presentation capacity. Given that this was only in a murine system, we wanted to examine the effect of repetitive stimulation of human CD141^+^ DCs with various immuno-potent lipids (e.g., MA, LAM, LPS, and PGN) every 48 h on IL-12 production. To our surprise, although IL-12 production was augmented as the number of stimulations with MA or LAM increased, treatment with either LPS or PGN only induced strong IL-12 secretion once. Moreover, repeated stimulation with LPS stopped the IL-12 secretion by DCs (Fig. 6j). These results suggest that repeated stimulation by MA or LAM induces continuous activation of DCs, while continued stimulation by LPS terminates activation.

## Discussion

Class I MHC molecules reflect various intracellular events, including virus replication and carcinogenesis, associated with gene conversion. The strongest effectors for recognizing the outcome of intracellular gene conversion in tumor cells and for achieving target tumor cell eradication have been thought to be class I MHC-restricted CD8^+^ CTLs. These CD8^+^ CTLs are regulated by unique DC subsets that express DEC-205 in mice or CD141 in humans, and each of these expressed molecules has potent cross-presenting capacity. Despite having a high potency in promoting anti-tumor responses, tumor-associated DCs are mostly defective in their functional activity and become tolerogenic with down-modulated co-stimulatory molecules [1]. Such tolerogenicity in neighboring DCs seems to be mediated by tumor-derived soluble factors. These include α-fetoprotein, VEGF, and TGF-β1 in murine hepatoma (Hepa1-6-1) cells [1] or CA125 and CA19-9 in human ovarian cancer cells [2]. Unfortunately, these factors suppress CTL-based immunity against tumors and permits their growth in vivo. Based on these findings, we concluded that tolerogenic DCs were the main cause of impaired CTL induction in vivo.

We recently reported that DEC-205^+^ DCs can be selectively activated in vivo by an intraperitoneal injection of α-GalCer, a specific ligand for CD1d lipid antigen-presenting molecules on DCs, in BALB/c mice [3]. In addition, we confirmed that α-GalCer selectively activated DEC-205^+^ DCs in spleen cells from B6 mice, and that the expression of co-stimulatory molecules (e.g., CD80, CD86, and CD40) on these DCs was upregulated and reached an optimal value at about 24 h after stimulation with α-GalCer [3]. These results led us to speculate that tolerogenic DCs within a tumor mass could be converted into functional immunogenic DCs, and we showed that this conversion could be mediated through sequential and repetitive α-GalCer administration. The DEC-205^+^ DCs with a cross-presenting capacity were converted into DCs with enhanced expression of co-stimulatory molecules and an augmented IL-12 secretion in vivo, with which naïve T cells within a tumor mass might be primed and activated in a murine model [3]. In this way, it was considered possible to activate tolerogenic DCs within the tumor mass through stimulation with an immuno-potent CD1-associated lipid/glycolipid, such as α-GalCer, to convert them into immunogenic DCs with sufficient expression of co-stimulatory molecules. We then speculated that converted immunogenic DCs could prime and activate tumor-specific CD8^+^ CTLs within a tumor mass and thereby control tumor growth, even in humans.

Intravesical administration with live BCG is considered a potent immunotherapy against human bladder carcinoma [28], so we examined whether BCG could selectively activate CD141^+^ DCs. We found that the proportion of CD141^+^ DCs was substantially increased among DCs treated with both live and heat-inactivated BCG. However, more than 60% of DCs were killed when incubated with 200 µg/mL of live BCG for 2 days and nearly 30% were killed when incubated with 200 µg/mL of heat-inactivated BCG, indicating that some BCG components are toxic to DCs. Additionally, various adverse events are reportedly associated with the presence of BCG subcomponents [23–25], suggesting that whole BCG, even when in an attenuated form, could be toxic to DCs. Thus, we must use more potent and non-toxic substances from BCG to activate CD141^+^ DCs. This led us to consider the effect of a purified non-toxic component from *M. tuberculosis* and BCG, such as MA, a potent molecule that may stimulate human DCs via the CD1b molecule [29, 30]. As shown in this study, CD141 expression on human DCs was more markedly enhanced when DCs were treated with 500 µg/mL of purified MA from the Aoyama B isolate than from the BCG isolate. Moreover, the purified MA was not at all toxic to DCs. In addition, CD141 expression was enhanced and CD1b expression was decreased when DCs were treated with 300 μg/mL of LAM. Together, we showed that purified MA (500 µg/mL) and LAM (300 μg/mL) from the Aoyama B isolate can be used as non-toxic materials to activate CD141^+^ DCs instead of whole *M. tuberculosis* or BCG.

Furthermore, co-stimulatory molecule expression on CD141^+^ DCs was enhanced, and MA and LAM synergistically enhanced CD141^+^, in particular XCR1^+^CD141^+^, expression on DCs and IL-12 secretion by DCs. These findings indicated that MA- and LAM-treated DCs may gain the ability to prime and activate antigen-specific CTLs. Based on these findings, we examined whether MA- and LAM-treated CD141^+^ DCs loaded with tumor antigen could prime naïve human autologous CD3^+^ T cells into tumor-specific CD8^+^ CTLs. Our results confirmed that this was possible in a class I MHC-restricted manner, and that repeat stimulation with MA and LAM induced continuous DCs activation. By contrast, stimulation with other lipids, such as LPS, appeared to terminate DC activation.

We conclude that our findings provide new insights into the tumor environment, indicating that there is a suppression of anti-tumor immunity by tolerogenic DCs, which is itself induced by tumor-derived factors. We show that these tolerogenic DCs can be converted to immunogenic DCs by repetitively stimulating them with the MA and LAM components of *M. tuberculosis*, and in turn, induce effective tumor-specific CTL-immunity in naïve T cells. Combination therapy with non-toxic MA and LAM from *M. tuberculosis* could be an option for inducing and prolonging the activation of tumor-specific CTLs in humans.

## Electronic supplementary material

Below is the link to the electronic supplementary material.

Supplementary material 1 (PDF 1088 kb)

## Abbreviations

7-AAD: 7-Amino-Actinomycin D
CCM: Complete culture medium
DEX: Dexamethasone
α-GalCer: α-Galactosylceramide
LAM: Lipoarabinomannan
MMC: Mitomycin-C
MDDCs: Monocyte-derived DCs
MA: Mycolic acid
PGN: Peptidoglycans
siRNA: Short interfering RNA
TLC: Thin-layer chromatography
