# Supplementary material for: Induction of tumor-specific CD8+ cytotoxic T lymphocytes from naïve human T cells by using Mycobacterium-derived mycolic acid and lipoarabinomannan-stimulated dendritic cells
Source: Cancer Immunol Immunother. 2019 Sep 17;68(10):1605–19. doi: 10.1007/s00262-019-02396-8 (PMC6805962; doi:10.1007/s00262-019-02396-8)
Supplement: Supplementary file 1 — Supplementary material 1 (PDF 1088 kb) [file 262_2019_2396_MOESM1_ESM.pdf]

Supplementary Fig.

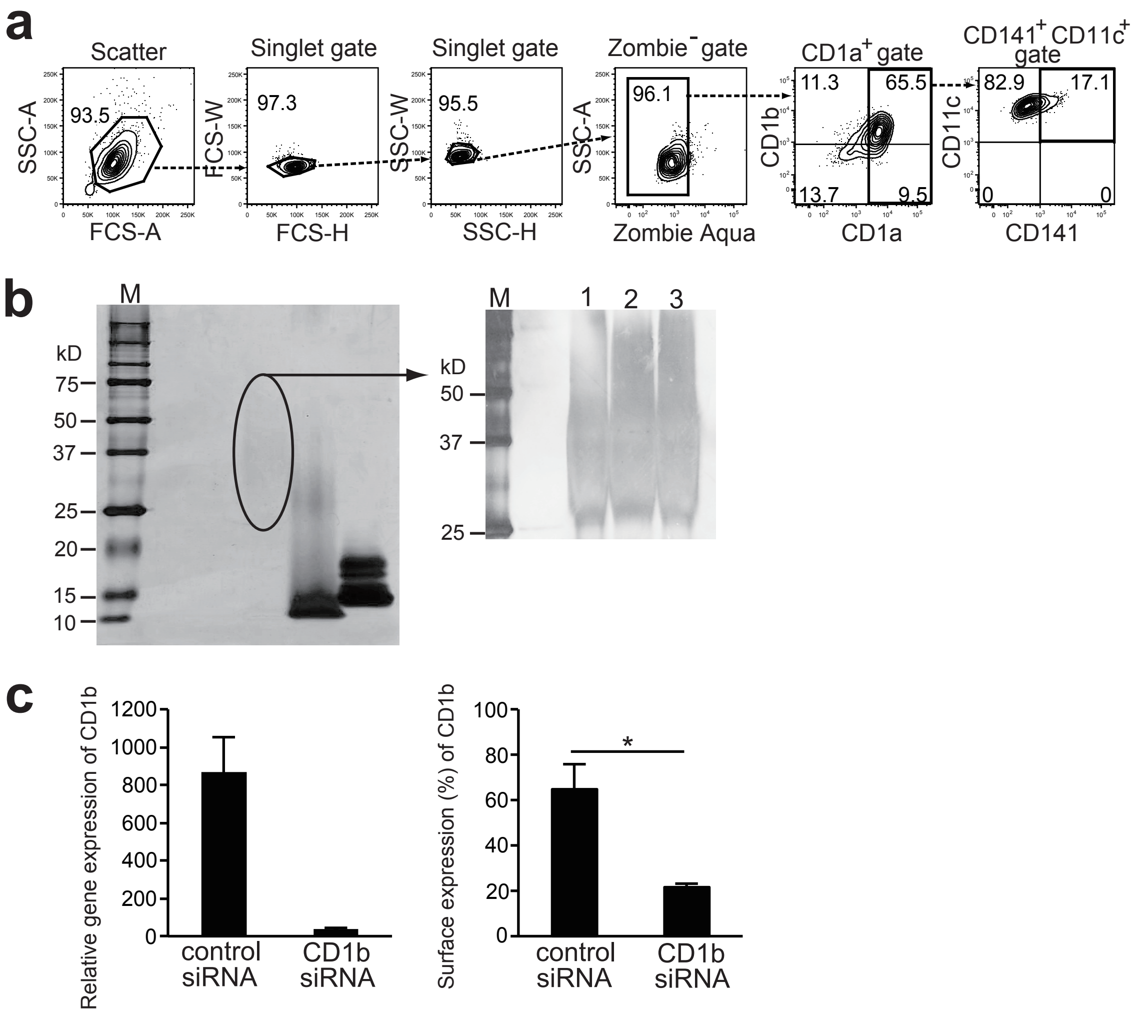

(a) Representative gating strategies of flow cytometry for the detection of DCs. DCs were identified as Zombie-, CD1a+, CD1b+, CD141+, and CD11c+.

(b) Temperature-dependency of protein detection in LAM by silver stain SDS-PAGE gel. Lane1: 100°C, 5min, Lane2: 70°C, 30 min, Lane3: 37°C, 2 hours. Lift figure is the same as Fig. 2a.

(c) Suppression of CD1b expression on MDDCs by using CD1b-siRNA. The left shows gene expression by real-time PCR and the right shows surface expression by flow cytometry.Date are shown as the mean ± S.D. of n=4 samples per measurement. *$p < 0.05$; one-way ANOVA analysis followed by Bonferroni post-tests to evaluate statistical differences.
